# 771. The impact of infectious diseases research involvement on future infectious disease fellowship application

**DOI:** 10.1093/ofid/ofad500.832

**Published:** 2023-11-27

**Authors:** Joseph Marcus, Heather Yun, Alice E Barsoumian

**Affiliations:** Brooke Army Medical Center, San Antonio, Texas; Brooke Army Medical Center, San Antonio, Texas; Brooke Army Medical Center, San Antonio, Texas

## Abstract

**Background:**

Infectious diseases physicians invest significant time mentoring medical students and internal medicine residents developing research projects as well as writing case reports. The impact of research or case reports on future application to infectious diseases fellowship is unknown.

**Methods:**

All research projects and case reports published or presented at conferences from Brooke Army Medical Center between 2014-2022 with an infectious diseases senior author and a medical student or internal medicine resident first author were evaluated. The presentations and publications that resulted from each project as well as whether the trainee applied to infectious diseases were recorded.

**Results:**

During the study period, 12 faculty mentored 51 trainees in 35 research projects and 26 case reports. Research and case reports were primarily performed by residents (88% and 96% respectively) (Table 1). Most trainees presented or published their research in multiple venues. Compared to case reports, research projects were more likely to be presented at national meetings (77% vs 31%, p=0.0003). Individuals who completed research projects had greater rates of infectious diseases fellowship application as compared to those who completed case reports (42% vs. 5%, p=0.006) (Table 2).

Summary of Research and Case Reports by Medical Students and Residents Mentored by Infectious Diseases Faculty at Brooke Army Medical Center, 2014-2022

**Figure:**
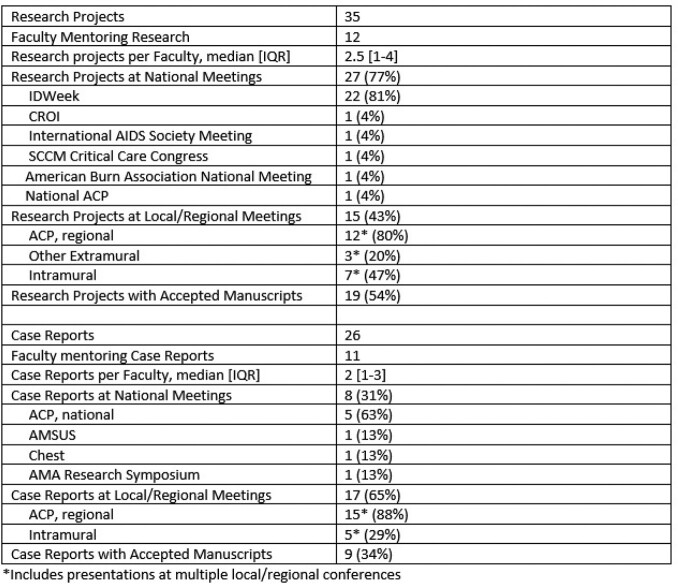

Internal Medicine Resident and Medical Student Application to Infectious Diseases Fellowship Organized by Projects Mentored by an Infectious Diseases Faculty Member at Brooke Army Medical Center, 2014-2022

**Figure:**
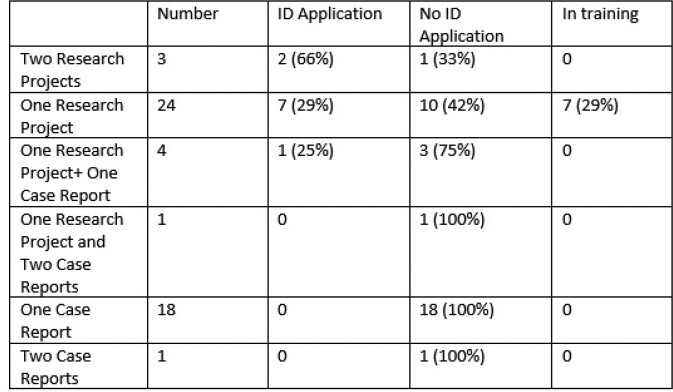

**Conclusion:**

Internal medicine resident and medical student involvement in research mentored by an infectious disease physician was associated with a higher infectious diseases fellowship application rate as compared to those who were mentored for case reports. Despite the significant time required to mentor trainees, investment in trainee research should be considered as an important component to the infectious diseases recruiting effort.

**Disclosures:**

**All Authors**: No reported disclosures

